# Making the invisible visible: integrated visualization and automated quantification of thrombus deformation during mechanical thrombectomy

**DOI:** 10.3389/fmedt.2026.1704010

**Published:** 2026-01-29

**Authors:** Marielle Ernst, Felizitas Sommer, Michael Bartl, Christian H. Riedel, Philip Langer

**Affiliations:** 1 Institute of Diagnostic and Interventional Neuroradiology, University Medical Center Göttingen, Göttingen, Germany; 2 Department of Neurology, University Medical Center Göttingen, Göttingen, Germany

**Keywords:** angiographic imaging, mechanical thrombectomy, segmentation, shape analysis, stroke, thrombus deformation, tracking

## Abstract

**Background:**

Mechanical thrombectomy using stent retrievers is a proven treatment for large vessel occlusion stroke, but quantitative and objective evaluation of device-thrombus interaction remains limited.

**Objective:**

This study introduces a novel algorithm and experimental framework to visualize and quantify thrombus deformation during retrieval under standardized *in vitro* conditions, with the long-term goal of informing future clinically applicable analysis tools.

**Methods:**

An *in vitro* model simulating large vessel occlusion was developed using organized human thrombi. Two stent retrievers - the Embotrap III (6.5 × 45 mm) and NeVa (4.5x 29 mm) - were studied in a vascular model under fluoroscopic guidance. An algorithm was developed to automatically segment and quantify thrombus deformation and analyze contour changes during the retrieval.

**Results:**

In total, 36 and 47 thrombectomies were performed with the Embotrap III and NeVa, respectively. Both devices achieved high first-pass reperfusion rates (Embotrap III: 88.9%, NeVa: 87.2%). However, thrombus deformation (mean: 14.7 × 10^−3^ vs. 8.9 × 10^−3^) and contour change (mean: 0.52 vs. 0.40) were significantly higher with Embotrap III, suggesting greater mechanical engagement. Thrombus fragmentation occurred in 5.6% of Embotrap III cases, but in none with NeVa. Moderate to marked distal thrombus migration occurred in over half of cases in both groups. Extensive migration occurred exclusively with NeVa in 4 cases (13%). Interrater and intrarater reliability of the automatic segmentation were excellent.

**Conclusions:**

Our new imaging and analysis framework allows for objective, reproducible measurements of thrombus-stent interaction over time. Our findings support the high efficacy of both stent retrievers. While the Embotrap III exerted stronger mechanical deformation effects on the clots, initial distal thrombus migration occurred more often with the NeVa device. This approach may support more informed device selection and optimization of thrombectomy strategies to enhance reperfusion success and reduce distal embolization in acute ischemic stroke.

## 1 Introduction

Ischemic stroke due to large vessel occlusion is a leading cause of long-term adult morbidity due to cognitive and functional impairment. Mechanical thrombectomy using stent retrievers has recently been established as a highly effective revascularization therapy for patients suffering from large vessel occlusion strokes (1).

While application of these devices has a common therapeutic goal, they vary in terms of structural design and mechanical properties, reflecting continuous technological advancements over the past decade (2).

Delivered via microcatheter across the clot, these devices deploy through gradual unsheathing, exerting radial force to compress the thrombus against the vessel wall and intercalate its struts into the clot structure (3).

Clot composition critically influences the mechanical interaction between thrombi and thrombectomy devices (4). Ischemic stroke clots are commonly classified as fibrin-rich or red blood cell-rich based on histology. Fibrin-rich clots contain dense, cross-linked fibrin-platelet networks, resulting in increased stiffness, elasticity, and resistance to deformation. In contrast, red blood cell-rich clots consist of erythrocytes within a looser fibrin matrix, leading to greater deformability and reduced vessel wall friction. These biomechanical differences affect device-clot integration, retrieval behavior, and recanalization outcomes. Especially fibrin-rich thrombi present a special challenge for revascularization devices (4).

Despite their clinical success, current methods used to evaluate stent retriever performance remain largely qualitative and subjective. Metrics such as first-pass reperfusion rates, number of retrieval attempts, distal embolization, and grading systems like the expanded Treatment in Cerebral Ischemia (eTICI) score offer valuable but indirect insights into device efficacy (5). However, these measures do not reveal the mechanical processes during the actual retrieval process.

In clinical practice, the interaction between the stent retriever and the thrombus occurs entirely out of view and cannot be directly observed with standard imaging modalities. The entire procedure is performed without visualizing the clot, since it is not radio-opaque and remains invisible during fluoroscopy. As a result, the precise mechanical events that determine success or failure of the performed retrieval remain in each thrombectomy maneuver unknown.

To improve understanding and comparison of device performance, there is a critical need for objective, quantitative methods that can characterize the interaction between stent retrievers and thrombi under controlled conditions.

Prior experimental and imaging studies suggest that the mechanical interaction between stent retrievers and thrombi, captured by parameters such as clot integration, deformation, and fragmentation, can be quantified (6–8). These parameters influence retrieval efficacy, fragmentation risk, and the likelihood of distal embolization, factors directly associated with patient outcomes after thrombectomy.

While prior studies have demonstrated that fluoroscopic data can be leveraged for quantitative assessment, they do not directly resolve thrombus geometry or deformation; instead, they rely on surrogate markers, such as deformations of the radiopaque stent retriever markers, to predict the location and boundaries of the invisible clot (9). Device-thrombus interaction metrics are not yet routinely assessed, and standardized, reproducible frameworks for their quantitative evaluation remain limited.

An important challenge lies in the operator dependence inherent in many imaging-based analyses. Variability in data acquisition, segmentation, and interpretation can introduce both inter- and intrarater differences, limiting the reproducibility and comparability of results across studies and institutions (10, 11). Establishing validated tools and standardized protocols that minimize observer variability is therefore essential to ensure reliability and facilitate widespread adoption of quantitative imaging methods in thrombectomy research.

In parallel, broader cardiovascular and perioperative fields emphasize systematic, quantitative approaches to complications and therapy monitoring (12, 13). These trends underscore the value of mechanistic, data-driven frameworks, to which our work contributes specifically in mechanical thrombectomy.

In this study, we aimed to visualize and quantitatively assess both the mechanical engagement between stent retrievers and thrombi, as well as the extent of thrombus deformation during retrieval, using a reproducible experimental setup.

Unlike previous studies that relied predominantly on qualitative assessment or limited quantitative metrics, the present work introduces an integrated experimental and image-analysis framework that makes the invisible device-thrombus interaction visible and automatically quantifies thrombus deformation, contour change and distal migration during stent retriever thrombectomy under standardized conditions. This approach may support the development of future clinically relevant visualization and analysis tools that could enhance understanding of stent-based thrombectomy mechanisms.

## 2 Materials and methods

To quantitatively evaluate the interaction between stent retrievers and thrombi, we developed an *in vitro* model designed to simulate large vessel occlusion and clot retrieval under controlled conditions.

### 2.1 Clot formation

Standardized thrombi were fabricated using human blood to achieve consistent mechanical properties and dimensions across trials.

Human whole blood was obtained from two healthy individuals (FS, ME). We utilized a modified Chandler loop technique to generate organized, firm thrombi (14, 15).

A silicone tubing with an inner diameter of 7 mm and a length of around 30 cm was fixed within a Chandler Loop System® (ebo kunze industriedesign, Neuffen, Germany). 0.12 mL of calcium chloride solution (1 N) were injected into the tubing. Following this, the tubing was filled with approximately 10 mL of citrated human whole blood, and the ends were connected to create a closed loop. The rotator device was set to rotate at a rate of 25 rotations per minute for one hour generating accelerated blood flow in order to simulate pulsatile carotid blood flow at a rate of approximately 240 mL/min.

Post-formation, thrombi underwent visual inspection for integrity, and uniformity to ensure only standardized clots advanced to testing.

### 2.2 Retrieval and image acquisition

To replicate the terminal segment of the internal carotid artery, we printed a 3D model featuring an internal lumen with a diameter of 5 mm and an internal recess measuring 4 mm in diameter. A three-way stopcock was integrated at each end. A thrombus was introduced into the recess and contrast medium was injected from both ends of the model. The contrast agent (Imeron®, 400 mg iodine/mL) was diluted in a ratio of 2:3 with sodium chloride solution (NaCl), yielding a two-thirds dilution. The 3D model was fixed on the angiosuite table to ensure stability during the retrieval.

A diagnostic catheter (6F) and microcatheter (Trevo Pro 18, Stryker, Kalamazoo, CA, USA) were introduced through the proximal luminal aperture. Subsequently, a single shot image was taken. Under fluoroscopically guidance, the microcatheter passed the site of occlusion, followed by another single shot image.

A stent retriever was then placed primarily distally to and with the proximal third across the occlusion site by using the active push deployment technique (16, 17). After stent retriever deployment another single shot image was taken. The retrieval was performed while acquiring fluoroscopic x-ray images at six frames per second with a spatial resolution of 1024 × 1024 pixels.

After the retrieval, a final single shot image was acquired. If necessary, the retrieval manoeuvre was repeated.

Two stent retrievers were used: the NeVa thrombectomy device (Vesalio LLC, Nashville, USA) in 4.5 × 29 mm with 3 drop zones, and the Embotrap^TM^ III revascularization device (Cerenovus, Johnson & Johnson Medical Devices, Irvine, California, USA) in 6.5 × 45 mm. Both devices are 0.021 in microcatheter compatible.

All experiments were repeated under identical conditions to ensure reproducibility.

### 2.3 Image analysis

Due to the high dimensionality of the acquired x-ray imaging data (1024 × 1024 × timestamps), a python 3.10 program was designed to streamline downstream processing, enhance runtime efficiency, and ensure a manageable timeframe for completion (3D - O(n^3^)). In order to achieve this, the program employed a manual selection process for a 2D region of interest (ROI) by the physician. This involved selecting a rectangular box around the ROI, representing the above-mentioned 3D printed vessel model. Given that the 3D print remains stationary while only its contents change during the thrombectomy, the selected rectangle remains valid across all timestamps. This manual input helped to reduce image complexity along both the X- and *Y*-axis dimensions, eliminating the need for a complex automated vessel-detecting algorithm. Another point selection in the middle of the vessel saved another complex implementation of an automated vessel-detecting algorithm.

After the physician's manual input, the automated part of the program took over by cropping the image to the selected area, reducing the pixels by approximately 80%. A median image was then generated over all images of the series, removing moving parts and leaving only the blank model without any interior. This median image served as a subtractable baseline image, enabling the normalization of every timestamp's image.

This normalization enables the use of a rudimentary thresholding algorithm for feature selection, highlighting the radiolucent thrombus, the radiopaque stent retriever tip, and other structures within the experimental setup. To further simplify the process for physicians, an algorithm allowed the physician to click and drag the mouse, evaluating touched pixels for minimum and maximum values during the drag. The pixels on the dragged path served as seed points for a 2D flood-fill algorithm, combined with thresholding by the encountered values mentioned earlier, enabling rapid and precise segmentation of ROIs within a single timestamps image. To further reduce manual intervention, the thresholding algorithm also operated on the temporal axis, applying the threshold to pixels on the next timestamp within the threshold interval. This process continued into both past and future timestamps until the end of the temporal series or until the ROI left the image frame.

Once all ROIs were marked up, a mask in Neuroimaging Informatics Technology Initiative (NIfTI) format was created, serving as the foundation for further investigation. This resulting NIfTI file is loaded into Slicer (version 4.20) for a final check by the physician, who corrected any errors during automated markup within the software.

To assess interrater reliability, a subset of 10 retrievals with the Embotrap III and 10 retrievals with the NeVa device was independently analyzed by a second rater who was blinded to the assessment of the first rater. To evaluate intrarater reliability, one rater repeated the image analysis of the same subset (*n* = 20) after a two-week interval to minimize recall bias. Segmentation variability was evaluated by comparing Dice similarity coefficient (DICE), Jaccard index (JACC), Hausdorff distance (HD95, mm), and average surface distance (ASD, mm) between raters and across sessions.

For statistical analysis of marked-up regions, the same Python program was called with modified startup parameters and the NIfTI mask as input. The program automatically calculated various parameters based on the original DICOM files' metadata pixel spacing (0028,0030) and the x-ray imaging series frame rate. This enabled translation of pixel data within the selected ROI into real world physical information, including traveled distance, speed, acceleration, shift from the middle axis, interpolated area, and uniformity of the physician's movement during stent retriever usage.

For evaluating the interaction between the stent retriever and the thrombus the following parameter were extracted:

- the Y-coordinates of both the stent retriever tip and distal thrombus tip. This enabled a quantitative comparison of the **stent retriever's grip** on the thrombus by determining the average change in distance between the stent tip and thrombus tip (in mm per slice) throughout the retrieval process. Distal thrombus migration was categorized based on the extend of displacement: minimal (<1 mm), moderate (≥1 mm to <2.5 mm), marked (≥2.5 mm to <4 mm), and extensive (≥4 mm).
- the thrombus deformation during the retrieval, measured as the sum of the absolute changes in the number of pixels per slice, divided by the initial number of pixels of the thrombus at the start of the retrieval, in order to account for differently sized thrombi.

Thrombus deformation is mathematically defined as:

Let:

- $\mathrm{sliceindex}\;\;i\in N_{0}\mathrm{with}\;0\leq i\leq \mathrm{slic}e_{\max}$
- $\mathrm{pixelcount}\;\mathrm{of}\;\mathrm{slice}\;\;0:p_{0}$
- $\mathrm{pixelcount}\;\mathrm{of}\;\mathrm{slice}\;\;i:p_{i}$

The thrombus deformation metric *D* is then defined as:

$$D=\frac{1}{N}\overset{N}{\underset{i=1}{\sum }}\frac{\left|\;p_{i}-p_{0}\right|}{\;p_{0}}$$

D is a dimensionless quantity representing the mean relative deformation during retrieval. For readability, reported values of *D* are upscaled by a factor of 10^3^.

- the **contour change** of the thrombus during the retrieval measured as a dimensionless numerical indicator of the average euclidean distance from each pixel of the contour to the nearest pixel of the thrombus’ outline on the next slice. In cases of no contour changes, the previous and current contour were equal, thus no changes were detected. Higher numbers indicate a stronger contour change.

Countour change is mathematically defined as:

Let $C_{t}=\{\;p_{i}=(x_{i},y_{i})|i=1,\ldots ,N\}$ be the set of *N* pixels on the contour at slice *t*, and $O_{t+1}$ the outline pixels at slice t+1. For each $p_{i}\in C_{t}$, compute the minimum Euclidean distance to $O_{t+1}$:

$$d(\;p_{i}\;,O_{t+1})=\;\underset{q\in O_{t+1}}{\min}\sqrt{{\left(x_{i}-x_{q}\right)}^{2}+{\left(y_{i}-y_{q}\right)}^{2}}$$

The contour change CC is the mean over all contour pixels:

$$CC=\frac{1}{N}\overset{N}{\underset{i=1}{\sum }}d(\;p_{i},O_{t+1})$$

This yields a dimensionless value (in pixels), as coordinates are in image pixel units; CC=0 when contours match exactly.

Error propagation in distance for one voxel equals 0.024 mm^2^ or 0.154 mm per side. The error for velocity for one voxel per frame at six frames per second equals 0.924 mm/s.

Descriptive statistics, including mean, median, standard deviation, 95% confidence interval and range, were calculated for all extracted parameters using R version 4.3.1. To assess differences in thrombus deformation and contour change between the two stent retriever devices, a non-parametric Wilcoxon rank sum test was conducted due to the non-normal distribution of values. The association between thrombus deformation and retrieval speed was also evaluated using a Wilcoxon rank-sum test. A *p*-value < 0.05 was considered statistically significant. For effect size cliff's δ was calculated.

An ANCOVA test was conducted to determine correlations between clot length and deformation or contour change across different devices. To assess the influence of device size on performance, data were stratified by device type (Embotrap III and NeVa). Subsequently, linear regression analyses (ANCOVA) were performed to examine the relationship between a comparable clot length and the measured parameters of deformation and contour change.

Colored temporal overlay images were generated by calculating the geometric center of gravity (GCOG) for each thrombus per timestamp. These GCOGs were then stacked to construct a composite image. To optimize alignment between slices, images were minimally shifted along the X- and Y-axes to maximize the overlap of Z-neighbouring thrombus segmentations. The resulting outlines were assigned a temporal color gradient, the hue ranging from red at the start of the retrieval to green at the end of the retrieval. Pixel brightness was scaled from 50% to 100%, corresponding to the degree of overlap at each location. A brightness of 50% indicated a pixel present in only one time point, while 100% indicated the maximum observed overlap (N pixels) across the retrieval. This allowed for a fast interpretation of the thrombus deformation over the complete time period of one extraction.

## 3 Results

A total of 36 thrombectomies were performed using Embotrap III with a first pass reperfusion in 32 cases (88.9%) and four failed retrievals. The thrombus was fragmented in two retrievals using Embotrap III.

With the NeVa stent retriever, 47 thrombectomies were performed with a first pass reperfusion in 41 cases (87.2%) and six failed retrievals; no thrombus was fragmented.

After quality assessment two thrombi that fragmented in the retrieval with an Embotrap III stent-retriever, were excluded. Nine retrievals with the NeVa stentretriever were excluded: four due to air emboli artifacts (i.e., imaging interference from air bubbles mimicking emboli in the vascular model under fluoroscopy) and five due to contrast issues (i.e., poor visualization from insufficient or uneven contrast enhancement).

**Figure 1:**
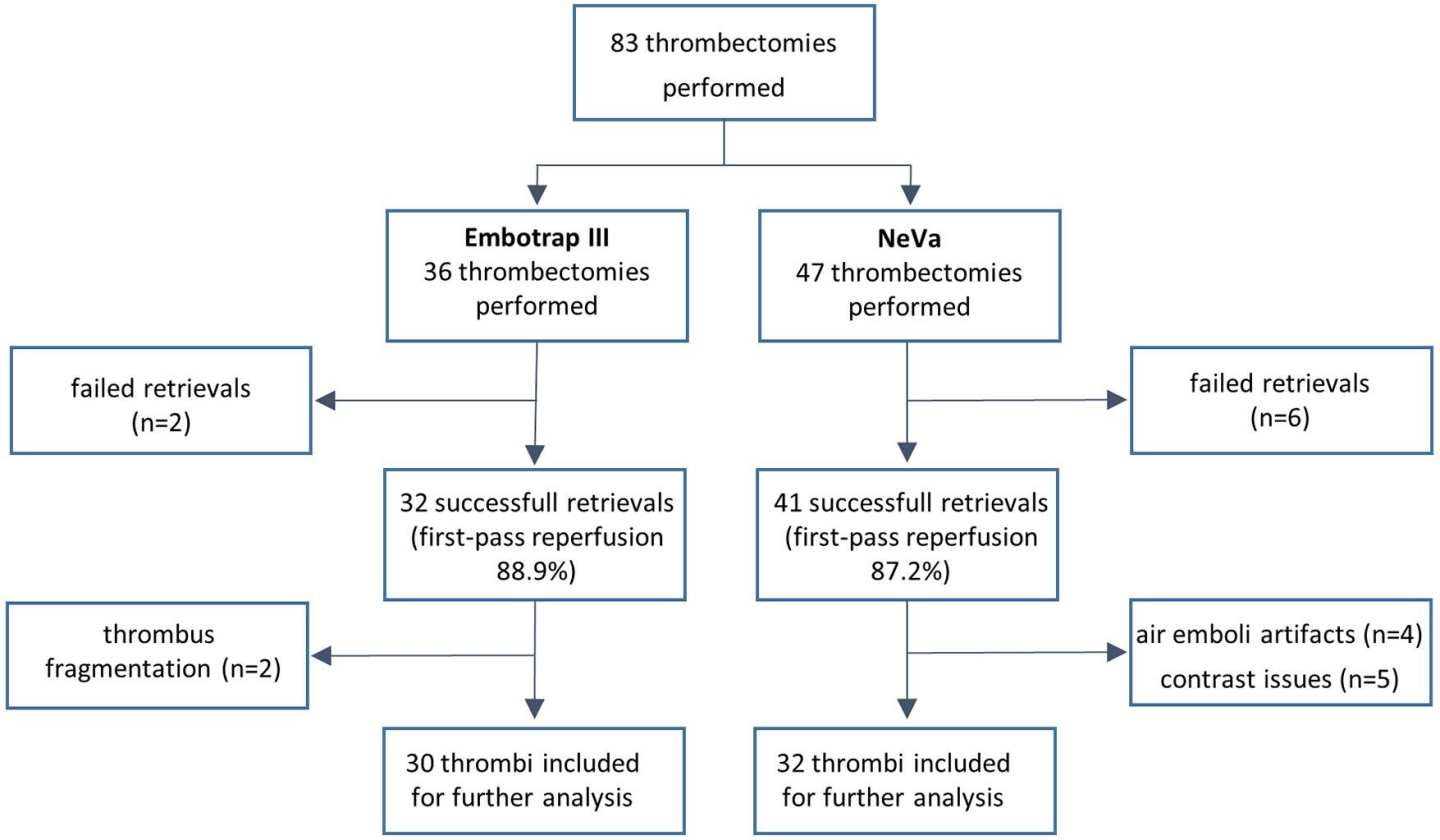
CONSORT flow diagram of thrombectomy procedures using Embotrap III and NeVa devices. This diagram illustrates participant flow through the study, starting from 83 total thrombectomies (36 with Embotrap III and 47 with NeVa). It details successful first-pass retrievals (32 for Embotrap III at 88.9% and 41 for NeVa at 87.2%), exclusions due to failed retrievals (2 with Embotrap III, 6 with NeVa), thrombus fragmentation (2 for Embotrap III), air emboli artifacts (4 for NeVa), and contrast issues (5 for NeVa), resulting in 32 and 30 thrombi included for further analysis, respectively.

Segmentation and further analysis of thrombus length, width, deformation and contour change were performed for the remaining 30 retrievals with the Embotrap III stentretriever and for 32 retrievals with the NeVa stent retriever ( Figure 1 ).

**Figure 2:**
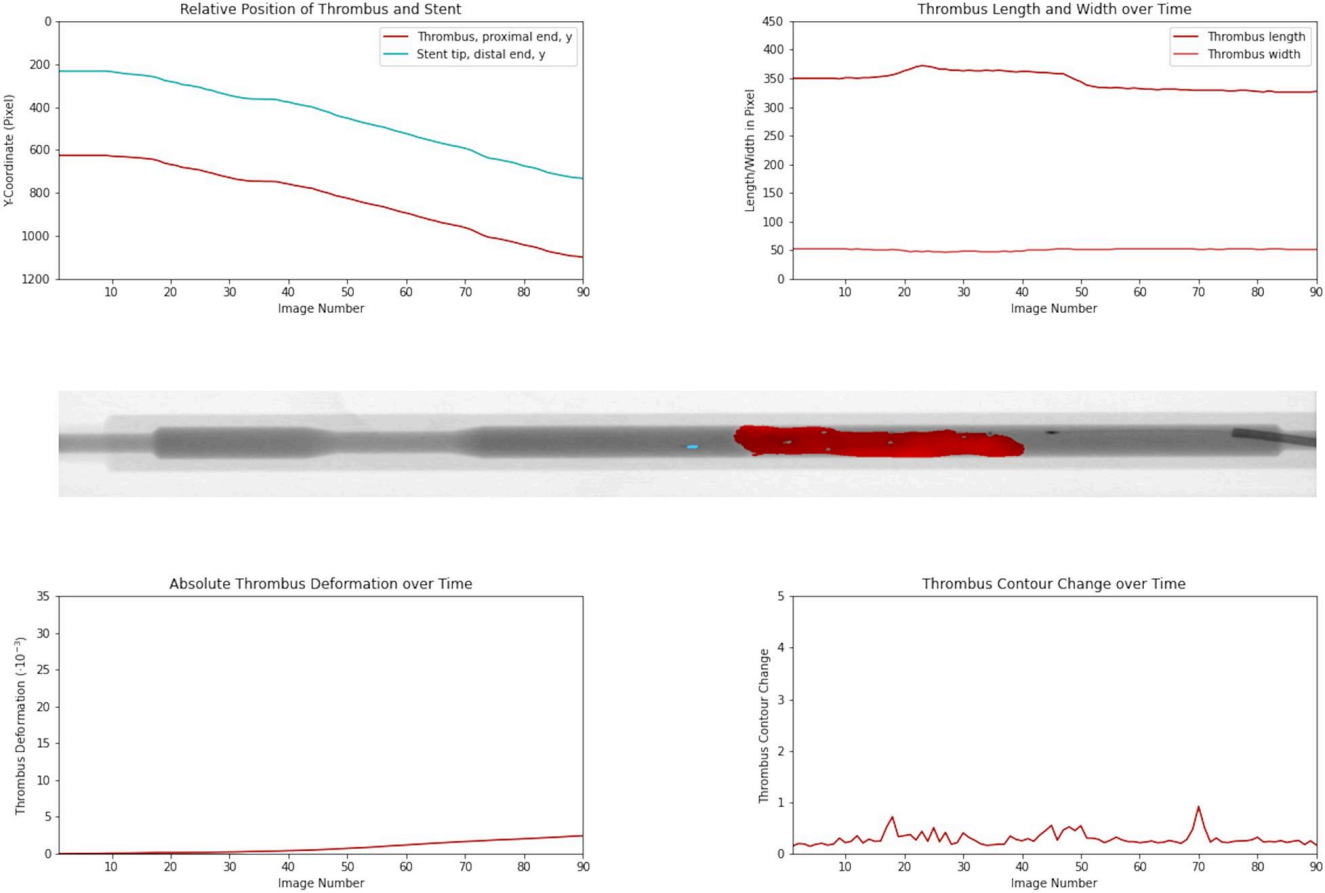
Temporal evolution of thrombus position, morphology, and deformation during successful retrieval. Top left panel shows Y-coordinates of the stent retriever tip and proximal thrombus tip across image frames, used to quantify changes in their relative distance. Top right panel shows the thrombus length and width over time. The middle panel illustrates an angiographic frame with thrombus segmentation (red). Bottom panels depict the absolute thrombus deformation (left) and contour change (right) throughout retrieval. The data exemplifies a successful retrieval with minimal thrombus distortion.

Thrombus deformation dynamics during successful retrieval with minimal distortion are illustrated in Figure 2 and Supplementary Video S1.

**Figure 3:**
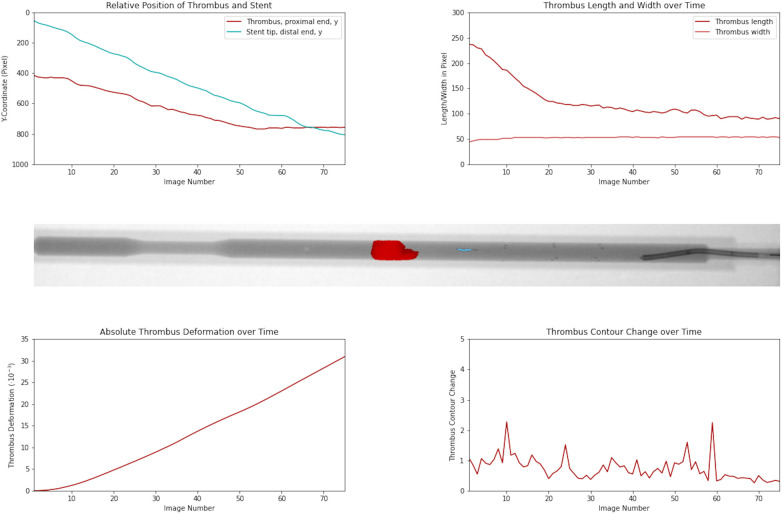
Temporal evolution of thrombus position, morphology, and deformation during failed retrieval. Top left panel shows Y-coordinates of the stent retriever tip and proximal thrombus tip across image frames, used to quantify changes in their relative distance. Top right panel shows the thrombus length and width over time. The middle panel illustrates an angiographic frame with thrombus segmentation (red). Bottom panels depict the absolute thrombus deformation (left) and contour change (right) throughout retrieval. The data exemplifies a failed retrieval with signiﬁcant deformation.

Figure 3 and Supplementary Video S2 show the time-resolved changes in thrombus length and deformation during retrieval, illustrating a failed retrieval associated with substantial deformation.

Regarding the **stent retriever's grip** on the thrombus during retrieval, minimal distal thrombus migration was observed in 12 cases (40%) with the Embotrap III stent retriever and in 11 cases (34%) with the NeVa stent retriever. An initial moderate distal migration followed by thrombus stabilization occurred in 14 cases (47%) with the Embotrap III and in 15 cases (47%) with the NeVa device. Marked initial distal migration was observed in 4 retrievals (13%) with the Embotrap III and in 2 retrievals (6%) with the NeVa. Notably, extensive distal migration occurred exclusively with the NeVa stent retriever in 4 cases (13%). The distribution of thrombus migration categories for both devices is summarized in the contingency analysis (Table 1). Fisher's exact test demonstrated no statistically significant difference in distal thrombus migration between the two stent retrievers (*p* = 0.242).

**Table 1:** Contingency table of migration categories for Embotrap III and NeVa devices.

Migration category	Embotrap III			NeVa		
	*n*	Share	CI_95%_	*n*	Share	CI_95%_
Minimal	12	0.400	[0.227–0.594]	11	0.344	[0.186–0.532]
Moderate	14	0.467	[0.283–0.657]	15	0.469	[0.291–0.653]
Marked	4	0.133	[0.038–0.307]	2	0.062	[0.008–0.208]
Extensive	0	0	–	4	0.125	[0.035–0.290]

CI, confidence interval. Fisher's exact test *p*-value 0.242.

**Figure 4:**
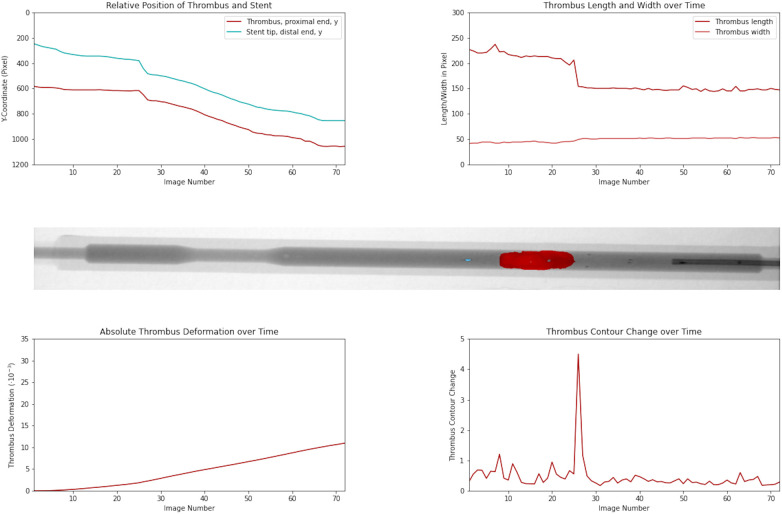
Distal thrombus migration during retrieval. The data demonstrates a retrieval characterized by an initial distal migration of the thrombus followed by stabilization of the thrombus.

Figure 4 and Supplementary Video S3 illustrate a retrieval process characterized by an initial distal migration of the thrombus, followed by its subsequent stabilization.

The **mean initial thrombus length** of the thrombus was 39 mm (±9 mm) in the Embotrap III group and 42 mm (±11 mm) in the NeVa group. The median initial thrombus length was 38 mm in the Embotrap III group and 41 mm in the NeVa group.

**Figure 5:**
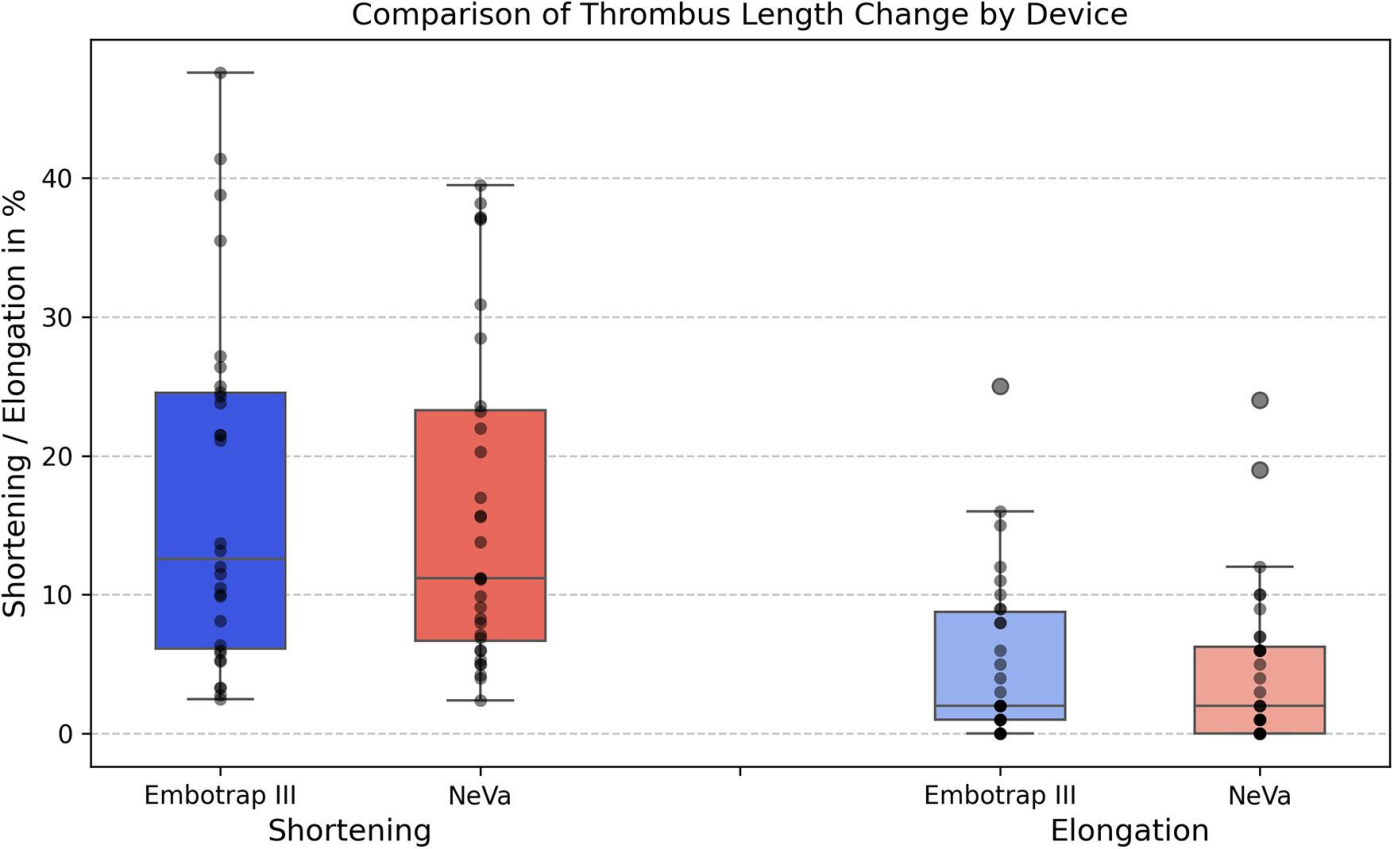
Comparison of thrombus length change by device. Box-and-whisker plots with individual case dots show percentage thrombus shortening and elongation relative to baseline length for the Embotrap III and NeVa stent retrievers. Boxes show the interquartile range, horizontal lines the median, whiskers 1.5 × interquartile range, and circles outliers. Group differences were analyzed using the Wilcoxon rank-sum test (Embotrap III, *N* = 30; NeVa, *N* = 32).

The mean **maximal thrombus shortening** during the retrieval was 6 mm (17%) ± 4 mm with the Emobtrap III stent retriever and 6 mm (16%) ± 5 mm with the NeVa stent retriever. The median maximal shortening was 5 mm (13%) with the Embotrap III stent-retriever and 5 mm (11%) with the NeVa stent-retriever. With the Embotrap III stent-retriever, the maximal shortening was 48%, with the NeVa stent-retriever 40%. Thrombus shortening of less than 10% of the initial thrombus were most frequent in both groups: *n* = 11 (37%) in the Embotrap III group, *n* = 14 (44%) in the NeVa group. The Embotrap-III-group had more cases with an elongation of 20%–30% (*n* = 10, 30%) and in two retrievals the shortening was >40% (Figure 5).

**Figure 6:**
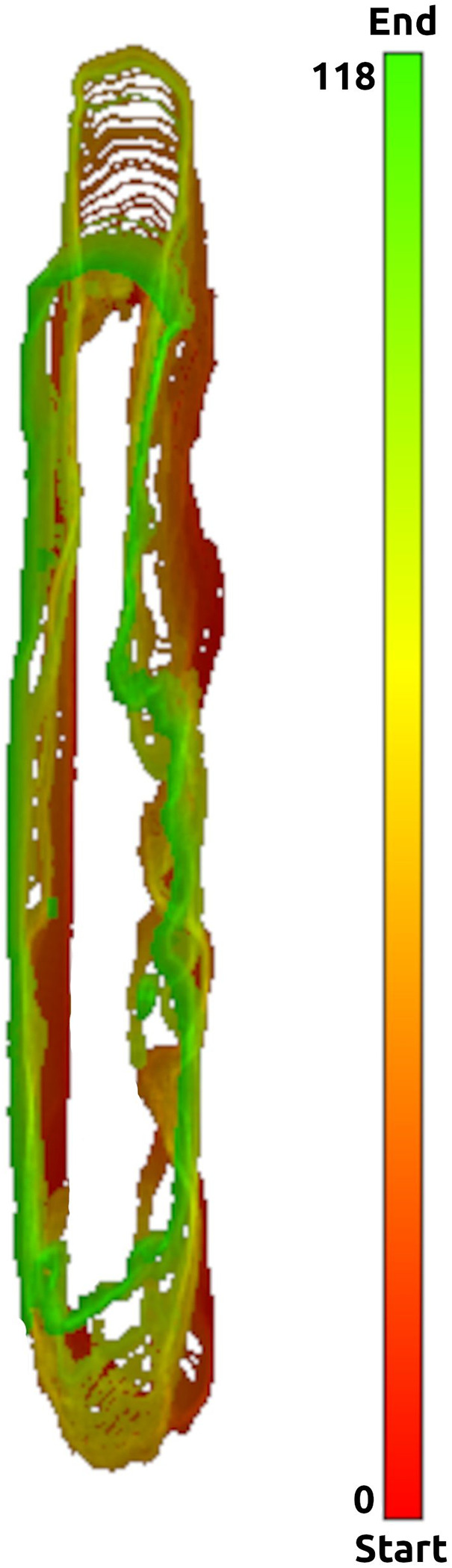
Colored temporal overlay images. Colored temporal overlay images illustrate thrombus deformation over time by stacking the geometric center of gravity, applying a hue gradient ranging from red (start) to green (end of retrieval) for temporal progression, and scaling pixel brightness (50%–100%) to indicate overlap frequency across time points.

The mean **maximal thrombus elongation** during the retrieval was 2 mm (5%) ± 3 mm with the Embotrap III stent retriever and 2 mm (5%) ± 3 mm with the NeVa stent-retriever. The median maximal elongation was 1 mm (2%) both with the Embotrap III stent-retriever and with the NeVa stent-retriever. With the Embotrap III stent-retriever, the maximal thrombus elongation was 25%, with the NeVa stent-retriever 24% (Figure 6).

Thrombus elongation of less than 10% of the initial thrombus was the most frequent in both groups (*n* = 24, 80% in the Embotrap III group, *n* = 28, 88% in the NeVa group). The Embotrap-III-group had more cases with an elongation of 10%–20% (*n* = 5, 17%).

**Figure 7:**
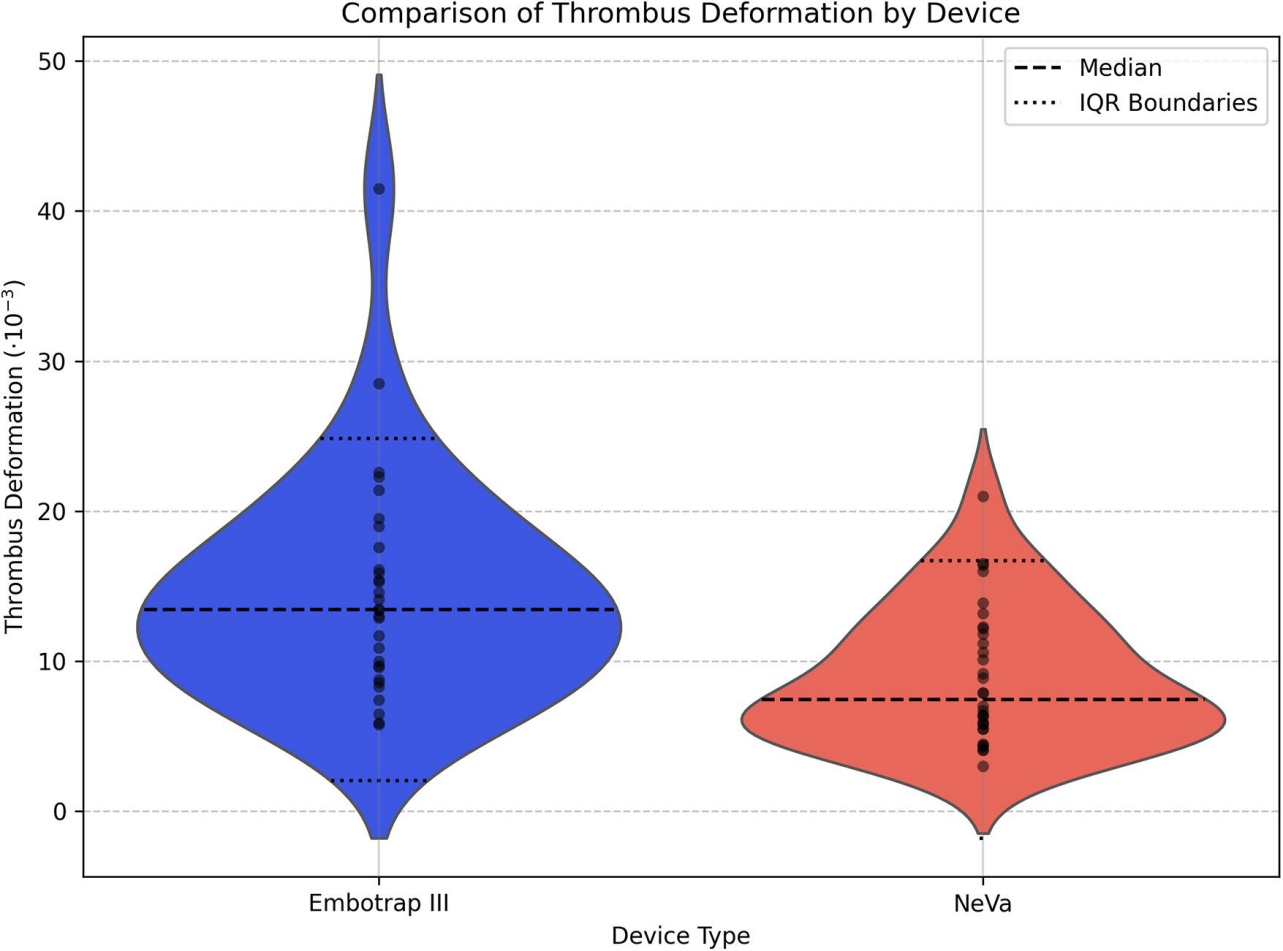
Comparison of thrombus deformation by device type**.** Violin plots with individual case dots show the distribution of thrombus deformation (dimensionless) during mechanical thrombectomy for the Embotrap III and NeVa stent-retriever devices. Dashed and dotted horizontal lines indicate the median and interquartile range boundaries, respectively. Thrombus deformation was compared between devices using the Wilcoxon rank-sum test (U = 735.5, *p* = 0.00033), with higher deformation observed in the Embotrap III group (mean 14.7 × 10^−3^, median 13.5 × 10^−3^) than in the NeVa group (mean 8.9 × 10^−3^, median 7.5 × 10^−3^); sample sizes were Embotrap III *n* = 30, and NeVa *n* = 32.

Concerning **thrombus deformation**, the Wilcoxon rank sum test showed a statistically significant difference between the Embotrap III group and the NeVa group (U-statistic = 735.5, *p*-value = 0.00033). The mean thrombus deformation during the retrieval was higher in the Embotrap III group (14.7 × 10^−3^) than in the NeVa group (8.9 × 10^−3^). The median thrombus deformation was also higher in the Embotrap III group (13.5 × 10^−3^) compared to the NeVa group (7.5 × 10^−3^) (Figure 7).

Concerning standard deviation, the Embotrap III group showed more variability (7.5 × 10^−3^) compared to the NeVa group (4.6 × 10^−3^).

Stratified descriptive statistics, significance testing, and effect sizes across thrombus migration categories are summarized in Table 2.

**Table 2:** Thrombus deformation with Embotrap III and NeVa: descriptive statistics, significance tests, and effect sizes across migration category.

Migration category	Embotrap III				NeVa				Significance and effect sizes	
	*n*	Mean (×10^−3^)	SD	CI_95%_	*n*	Mean (×10^−3^)	SD	CI_95%_	*p*-value (FDR-BH)	Cliff's δ
Minimal	12	14.655	9.102	±5.783	11	8.655	4.632	±3.074	0.074	0.492
Moderate	14	14.879	6.909	±4.064	15	9.380	5.244	±2.980	0.045*	0.538
Marked	4	13.925	5.465	±6.014	2	8.400	0.707	±1.101	0.133	1
Extensive	0	–	–	–	4	8.050	1.775	±1.953	–	–

CI, confidence interval; SD, standard deviation; FDR-BH, false discovery rate (Benjamini-Hochberg).

Statistical significance was assessed between devices within each migration category (Wilcoxon rank-sum test, FDR-BH-adjusted); statistically significant differences are denoted by an asterisk (*p* < 0.05). Effect size is reported as Cliff's delta (*δ*), where values closer to 1 indicate larger effects.

**Figure 8:**
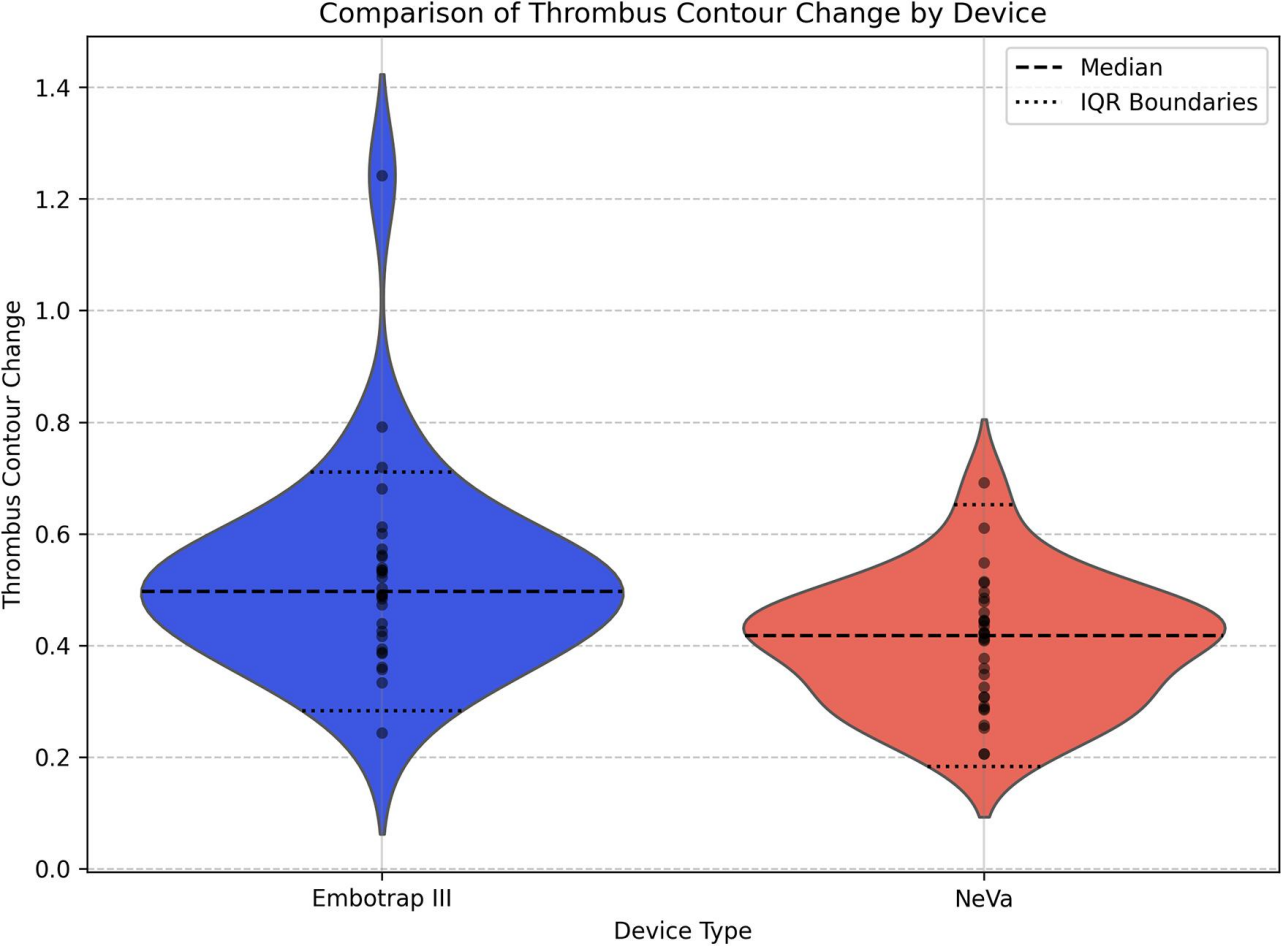
Comparison of thrombus contour change by device type. Violin plots show the distribution of thrombus contour change (dimensionless) during retrieval for the Embotrap III stent-retriever group (*N* = 30) and the NeVa stent-retriever group (*N* = 32). Per-case values are overlaid as individual points, and the dashed horizontal line in each violin denotes the group median, while the dotted lines indicate the interquartile range (IQR) boundaries. Group differences were assessed with the Wilcoxon rank-sum test (U = 688.0, *p* = 0.00133).

Concerning **contour change**, the Wilcoxon rank sum test showed a statistically significant difference between the Embotrap III group and the NeVa group (U statistic = 688.0, *p*-value = 0.00133). The mean contour change during the retrieval was higher in the Embotrap III group (0.52) than in the NeVa group (0.40). The median contour change was also higher in the Embotrap III group (0.50) compared to the NeVa group (0.42) (Figure 8).

Concerning standard deviation, the Embotrap III group showed more variability (0.18) compared to the NeVa group (0.11).

Stratified descriptive statistics, significance testing, and effect sizes across thrombus migration categories are summarized in Table 3.

**Table 3:** Mean contour change with Embotrap III and NeVa: descriptive statistics, significance tests, and effect sizes across migration category.

Migration category	Embotrap III				NeVa				Significance and effect sizes	
	*n*	Mean (×10^−3^)	SD	CI_95%_	*n*	Mean (×10^−3^)	SD	CI_95%_	*p*-value (FDR-BH)	Cliff's δ
Minimal	12	0.517	0.246	±0.156	11	0.361	0.083	±0.055	0.038*	0.591
Moderate	14	0.533	0.137	±0.081	15	0.407	0.140	±0.080	0.038*	0.495
Marked	4	0.504	0.079	±0.086	2	0.464	0.030	±0.047	0.533	0.5
Extensive	0	–	–	–	4	0.469	0.043	±0.047	–	–

CI, confidence interval; SD, standard deviation; FDR-BH, false discovery rate (Benjamini-Hochberg).

Statistical significance was assessed between devices within each migration category (Wilcoxon rank-sum test, FDR-BH-adjusted); statistically significant differences are denoted by an asterisk (*p* < 0.05). Effect size is reported as Cliff's delta (*δ*), where values closer to 1 indicate larger effects.

Linear regression analyses (ANCOVA) indicated no significant correlation between thrombus length and either deformation or contour change within each device group:

- Embotrap III: deformation (*p* = 0.143), contour change (*p* = 0.461)
- NeVa: deformation (*p* = 0.359), contour change (*p* = 0.872)

Mean retrieval speed with Embotrap was 5.704 mm/s, SD: 1.010 mm/s, CI: ±0.497 mm/s. Mean retrieval speed with Neva was 5.550 mm/s, SD: 2.315 mm/s, CI: ±0.999 mm/s. No significant correlation was observed between deformation and retrieval speed (Wilcoxon rank-sum test, *p* = 0.12).

Interrater and intrarater reliability of the automatic segmentation were excellent, with high spatial overlap (DICE, JACC) and low boundary error (HD95, ASD) for both devices. Quantitatively, metrics were slightly better for intrarater than interrater comparisons.

### 3.1 Interrater reliability

Embotrap III: Mean DICE 0.94 and JACC 0.88, with mean HD95 0.57 mm and ASD 0.11 mm, indicating very high agreement between raters and minimal segmentation boundary deviation.

NeVa: Mean DICE 0.95 and JACC 0.91, with mean HD95 0.56 mm and ASD 0.09 mm, likewise demonstrating excellent interrater concordance.

### 3.2 Intrarater reliability

Embotrap III: Mean DICE 0.99 and JACC 0.98, with mean HD95 0.06 mm and ASD 0.02 mm, reflecting near-perfect repeatability of the same rater's segmentations.

NeVa: Mean DICE 0.99 and JACC 0.98, with mean HD95 0.07 mm and ASD 0.02 mm, confirming similarly excellent intrarater agreement.

**Figure 9:**
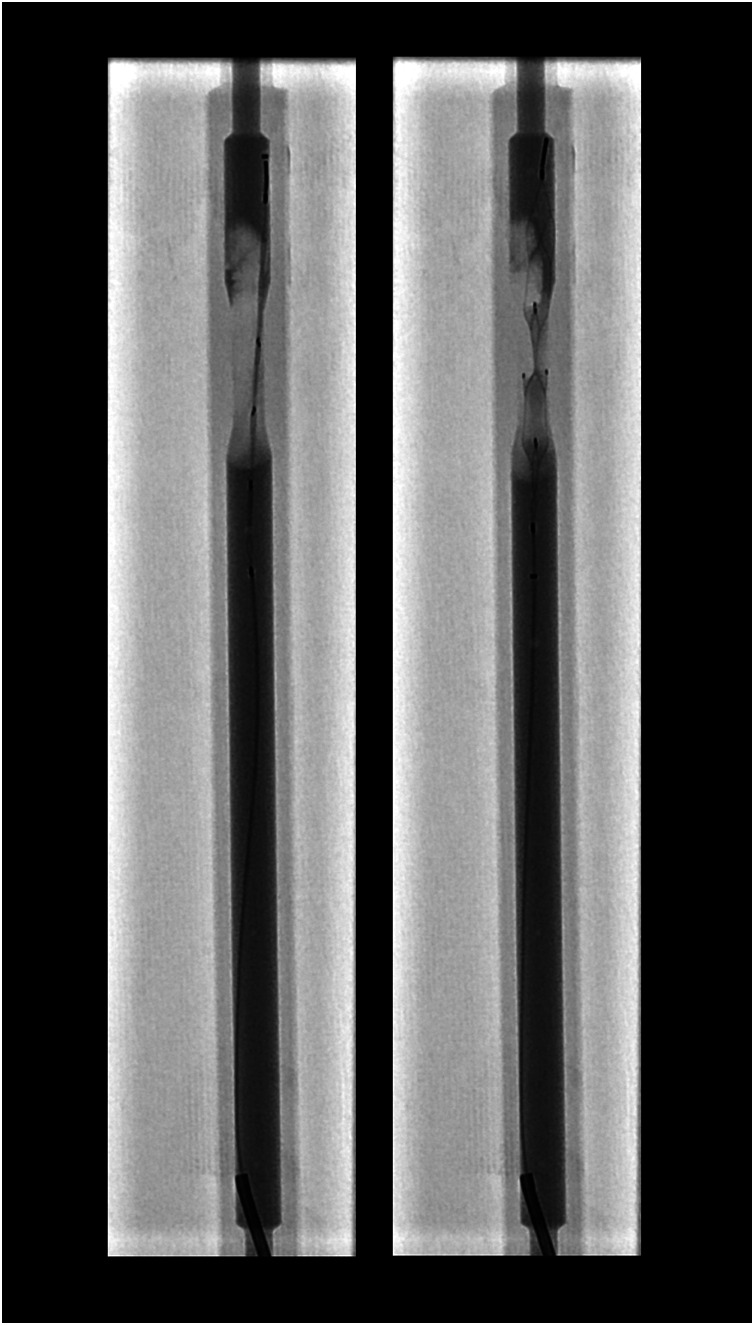
Peripheral microcatheter navigation and thrombus displacement. x-ray illustrating the typical advancement path of the microcatheter along the periphery of the thrombus, rather than through its core. Upon retraction of the microcatheter and deployment of the stent retriever, the thrombus is predominantly displaced against the vessel wall.

A noteworthy observation was that, in nearly all cases, the microcatheter did not pass through the thrombus but rather advanced along its periphery. Upon deployment of the Stent retriever by retracting the microcatheter, the thrombus was predominantly displaced against the wall (Figure 9).

Statistical analyses are summarized in Table 4 (Statistical Summary).

**Table 4:** Statistical summary.

Parameter	Embotrap III	NeVa	Statistical comparison
First-pass reperfusion, *n*/*N* (%)	32/36 (88.9%)	41/47 (87.2%)	Comparable
Thrombus deformation (×10 ^−3^ )	Mean ± SD: 14.7 ± 7.5 CI_95%_: ±2.7 Range: 5.8–41.5	Mean ± SD: 8.9 ± 4.6 CI_95%_: ±1.6 Range: 3.0–21.0	*p* = 0.00033*
Contour change (mean)	0.52	0.40	*p* = 0.00133*
Mean maximal thrombus shortening/elongation (mm)	6 ± 4	6 ± 5	
Segmentation reliability	Excellent (DICE 0.94–0.99)	Excellent (DICE 0.95–0.99)	Comparable

CI, confidence interval; DICE, Dice similarity coefficient; SD, standard deviation.

Statistically significant differences are denoted by an asterisk (*p* < 0.01; Wilcoxon rank sum test).

## 4 Discussion

In our study, we developed an algorithm to visualize and quantitatively measure both the interaction between the stent retriever and thrombus, as well as the degree of thrombus deformation during retrieval, under reproducible and objective conditions. While in clinical practice the interaction between the stent retriever and thrombus is not visible, our approach provided valuable insights into what actually occurs to the thrombus during mechanical thrombectomy. This visualization method enabled a deeper understanding of the mechanical dynamics at play, which could inform future strategies to optimize thrombectomy techniques and improve patient outcomes.

Earlier *in-vitro* studies of thrombectomy devices primarily focused on retrieval forces, clot capture success, and gross embolization patterns, with only limited geometric characterization of the clot using surrogate markers like deformations on the radiopaque stent retriever markers (6, 9).

This study is, to our knowledge, the first to combine a reproducible *in vitro* large-vessel occlusion model using human thrombi with an automated algorithm capable of tracking and quantifying thrombus deformation and contour changes throughout the entire retrieval sequence.

The results of this study present a detailed comparison between the Embotrap III and NeVa stent retrievers, focusing on key aspects such as first-pass reperfusion success, thrombus deformation, shortening, elongation, and contour changes during retrieval. Additionally, thrombus fragmentation and the behavior of the microcatheter in navigating the thrombus were also analyzed.

### 4.1 Thrombus composition

As has been shown previously, the performance of thrombectomy devices is inﬂuenced by the composition of the occluding thrombus (4). In comparison to thrombi rich in red blood cells, organized and firm thrombi, which are typically fibrin-rich, are more resistant to thrombolysis and present a special challenge for revascularization devices (4). We therefore focused on these organized thrombi formed using the Chandler loop technique. Our observation that the microcatheter did not pass through the thrombus but instead advanced along its periphery confirms the higher stiffness of our organized thrombus.

### 4.2 Chosen stent retriever

Moreover, we directed our attention toward two novel stent retrievers, NeVa and Embotrap III, both of which feature interesting designs for capturing thrombi.

The NeVa thrombectomy device is a stent retriever designed to capture organized thrombi *en bloc* within the central scaffold through openings in the stent structure known as drop zones.

The Embotrap III revascularization device is a dual layer stent-retriever that is designed to simultaneously restore ﬂow and entrap and remove the occlusive thrombus. The segmented design aims to improve vessel wall apposition of the stent retriever, and a distal filter is intended to capture potential clot fragments during the retrieval process.

There are no clinical studies comparing the NeVa stent retriever to the Embotrap III revascularization device. In the recent CLEAR study (18) the NeVa demonstrated superior first pass success compared with a predicate performance goal based on two studies of the EmboTrap (ARISE II study) and Tigertriever (TIGER study) devices.

### 4.3 First-pass reperfusion

In our study, both the Embotrap III and NeVa stent retrievers demonstrated high rates of first-pass reperfusion, with 88.9% and 87.2% success rates, respectively. These results align with previous studies showing that modern stent retrievers offer effective reperfusion rates in acute ischemic stroke management. Despite the comparable overall first pass reperfusion rates in our data, the procedural outcomes revealed subtle differences between the two devices.

### 4.4 Thrombus fragmentation

Notably, thrombus fragmentation occurred in two cases (5.6%) using the Embotrap III, whereas no fragmentation was observed with the NeVa stent retriever. Fragmentation can lead to the formation of smaller emboli, increasing the risk of distal ischemia or requiring additional interventions. The lack of fragmentation observed with the NeVa stent retriever may indicate a more controlled interaction with the thrombus, potentially minimizing the risk of distal embolization and improving the overall safety profile of the device in specific cases.

The slightly higher fragmentation rate observed with the Embotrap III may be explained by its larger stent diameter (6.5 mm vs. 4.5 mm for NeVa) and stronger mechanical engagement, as reflected by the significantly higher deformation and contour change values measured in this study. These characteristics likely produce greater compressive and shear forces during retrieval, which enhance thrombus capture but may simultaneously increase localized stress on less cohesive regions of the thrombus, leading to partial structural disruption.

The results of the ANCOVA test showed no significant correlation between thrombus length and either deformation or contour change within each device group. These results suggest that the measured device performance parameters were independent of the clot size and, consequently, of the device size itself for both device types.

### 4.5 Thrombus shortening and elongation

Both devices showed similar maximal thrombus shortening during retrieval - mean 6 mm (17%) for Embotrap III and 6 mm (16%) for NeVa - with maximal shortening reaching 48% and 40%, respectively. Elongation was minimal and comparable between devices (mean 2 mm, 5% in both groups), with most cases showing elongation below 10%.

### 4.6 Thrombus deformation and contour change

A notable difference between the two devices was observed in terms of thrombus deformation and contour change. The Embotrap III stent retriever induced significantly more deformation and contour change compared to the NeVa stent retriever. The mean deformation was 14.7 × 10^−3^ for the Embotrap III and 8.9 × 10^−3^ for the NeVa, while the mean contour change was 0.52 for the Embotrap III and 0.40 for the NeVa. These differences were statistically significant, suggesting that the Embotrap III stent retriever exerts more mechanical force on the thrombus during retrieval. This could be advantageous in cases where the thrombus is resistant or particularly cohesive. One possible explanation is the larger diameter of the Embotrap III stent retriever compared to that of the NeVa device.

### 4.7 Distal thrombus migration

Minimal distal thrombus migration, which can be considered as a favorable indicator of thrombus engagement, was seen in approximately one-third of retrievals with both devices. Moderate to marked distal thrombus migration occurred in more than half of the cases in both the Embotrap III and NeVa groups, underscoring the necessity of deploying the stent retriever primarily distally to and with the proximal third across the occlusion site.

The greater thrombus deformation induced by the Embotrap III might also explain the finding that distal thrombus migration during retrieval was observed less frequently, while extensive distal migration occurred exclusively with the NeVa device (13%).

### 4.8 Inter- and intrarater reliability

The inter- and intrarater analyses demonstrate that the automatic segmentation workflow is highly robust, with excellent spatial agreement and very small boundary deviations for both stent retriever types. These findings indicate that operator dependence and segmentation bias are unlikely to have materially influenced the reported quantitative metrics, as shown by consistently high DICE and JACC values and low HD95 and ASD across raters and time points. Consequently, the observed differences in clot morphology and device performance in this study can be interpreted with greater confidence, since they are not primarily driven by variability in the segmentation process.

## 5 Limitations

The authors acknowledge that some limitations to this study exist that merit consideration; in particular, only fibrin rich thrombi were investigated. RBC rich thrombi are prone to fragmentation and bear a higher danger of distal emboli (19). Thus, the frequency of thrombus fragmentation and the occurrence or prevention of distal embolisms could vary, especially as we did not use a flow model. These findings should therefore primarily be interpreted in the context of fibrin-rich, organized thrombi. RBC-rich clots, being softer and less cohesive, may display different deformation and retrieval behavior, potentially accentuating device-specific mechanical effects.

*In vitro* and *in vivo* thrombectomy models provide complementary information. *In vitro* phantoms enable standardized, reproducible evaluation of device-clot interactions and recanalization mechanics but cannot reproduce biological responses. In contrast, *in vivo* animal models offer greater physiological realism and safety assessment, yet differ anatomically from human vasculature and are subject to ethical and inter-subject variability (8). Thus, our rigid straight 3D model does not account for the curvature of cranial vessels, thereby neglecting the benefit of the segmented design featured in the Embotrap III, which enhances the stent retriever's ability to adhere to the vessel wall in curved vascular segments. Vessel curvature in real-life conditions could alter stent-vessel interaction and clot engagement dynamics, possibly leading to differences in deformation and distal migration behavior. However, with our model motion artefacts could be excluded, which made an automated objective quantitative image analysis possible.

Another limitation is that our model did not simulate physiological blood flow. However, this flow-free setup can still be considered clinically relevant, as current thrombectomy guidelines recommend the use of balloon guide catheters to achieve proximal flow arrest during retrieval and reduce embolization in new territories (20). This technique effectively creates a transient flow-free environment similar to our experimental conditions, thereby allowing isolation of mechanical device-thrombus interactions without the confounding effects of hemodynamic forces.

Finally, we cannot account for iatrogenic endothelial injury after stent retriever thrombectomy that has been demonstrated in previous studies (21, 22).

However, we did not aim to compare two stent-retriever in clinical routine but with regard to their interaction and deformation of the thrombus. The ability to quantitatively and objectively measure a dynamic process such as the deformation of a complex structure over time allows the evaluation of mechanical thrombectomy devices in a preclinical environment.

## 6 Future directions

Future studies could expand on our findings by analyzing the performance of different stent retriever designs across a broader range of thrombus characteristics and vessel sizes. In particular, simulating occlusions in smaller or more distal vessels could provide valuable insights into device behavior in anatomically challenging conditions.

Moreover, the future potential of our framework lays in the efficient and consistent ability to segment a large amount of retrievals. This enables the training of artificial intelligence for real-time segmentation during live-retrieval to evaluate possible complications during and after mechanical thrombectomy. It may even be possible to base a retriever recommendation on the previously segmented thrombus, which may lead to more effective reperfusion, lesser complications, and an increased rate of survival.

## 7 Conclusion

In conclusion, our novel algorithm and experimental setup to visualize and objectively measure thrombus-stent interaction during the retrieval has provided valuable insights into the mechanical processes involved in thrombectomy. Our findings support the high efficacy of both the Embotrap III and NeVa stent retrievers in achieving first pass reperfusion. In comparison, the Embotrap III stent retriever (6.5 × 45 mm) induced significantly more thrombus deformation and contour change compared to the NeVa stent retriever (4.5 × 29 mm), while initial distal thrombus migration occurred more often with the NeVa device. The standardized and reproducible nature of our framework, supported by excellent inter- and intrarater reliability of the semi-automatic segmentation, enables objective, quantitative evaluation of stent-thrombus interactions, and strengthens confidence in the robustness of the reported metrics. This approach offers a foundation for systematic assessment of design parameters influencing device performance and may guide the optimization of stent retriever geometry, material properties, and procedural strategies to improve clinical outcomes.

## Data Availability

The raw data supporting the conclusions of this article will be made available by the authors, without undue reservation.
