# Correction: Making the invisible visible: integrated visualization and automated quantification of thrombus deformation during mechanical thrombectomy

**DOI:** 10.3389/fmedt.2026.1799255

**Published:** 2026-03-17

**Authors:** Marielle Ernst, Felizitas Sommer, Michael Bartl, Christian H. Riedel, Philip Langer

**Affiliations:** 1 Institute of Diagnostic and Interventional Neuroradiology, University Medical Center Göttingen, Göttingen, Germany; 2 Department of Neurology, University Medical Center Göttingen, Göttingen, Germany

**Keywords:** angiographic imaging, mechanical thrombectomy, segmentation, shape analysis, stroke, thrombus deformation, tracking

The funder Deutsche Forschungsgemeinschaft (DFG—413501650) and Else Kröner-Fresenius-Stiftung (2025_EKEA.03), awarded to Michael Bartl, was erroneously omitted from the funding statement. The corrected funding statement appears below:

“The author(s) declared that financial support was received for this work and/or its publication. This work was supported by the Deutsche Forschungsgemeinschaft (DFG—413501650) and Else Kröner-Fresenius-Stiftung (2025_EKEA.03), awarded to MB”.

The original version of this article has been updated.

